# VHL-deficiency leads to reductive stress in renal cells

**DOI:** 10.1016/j.freeradbiomed.2023.07.029

**Published:** 2023-11-01

**Authors:** Hans Vellama, Kattri-Liis Eskla, Hillar Eichelmann, Andria Hüva, Daniel A. Tennant, Alpesh Thakker, Jennie Roberts, Toomas Jagomäe, Rando Porosk, Agu Laisk, Vello Oja, Heikko Rämma, Vallo Volke, Eero Vasar, Hendrik Luuk

**Affiliations:** a Institute of Biomedicine and Translational Medicine, Department of Physiology, University of Tartu, Tartu, Estonia; b Centre of Excellence for Genomics and Translational Medicine, University of Tartu, Tartu, Estonia; c Institute of Biomedicine and Translational Medicine, Department of Pathophysiology, University of Tartu, Tartu, Estonia; d Institute of Technology, University of Tartu, Tartu, Estonia; e Institute of Metabolism and Systems Research, College of Medical and Dental Sciences, University of Birmingham, Birmingham, UK; f Institute of Biomedicine and Translational Medicine, Department of Biochemistry, University of Tartu, Tartu, Estonia

**Keywords:** Reductive stress, Renal cell carcinoma, VHL syndrome, VHL, HIF, Respiration

## Abstract

Heritable renal cancer syndromes (RCS) are associated with numerous chromosomal alterations including inactivating mutations in von Hippel-Lindau (VHL) gene. Here we identify a novel aspect of the phenotype in VHL-deficient human renal cells. We call it reductive stress as it is characterised by increased NADH/NAD^+^ ratio that is associated with impaired cellular respiration, impaired CAC activity, upregulation of reductive carboxylation of glutamine and accumulation of lipid droplets in VHL-deficient cells. Reductive stress was mitigated by glucose depletion and supplementation with pyruvate or resazurin, a redox-reactive agent. This study demonstrates for the first time that reductive stress is a part of the phenotype associated with VHL-deficiency in renal cells and indicates that the reversal of reductive stress can augment respiratory activity and CAC activity, suggesting a strategy for altering the metabolic profile of VHL-deficient tumours.

## Highlights

• Reductive stress is evidenced by increased NADH/NAD^+^ and reduced respiration in VHL-deficient cells.
• Glucose removal and pyruvate/resazurin addition relieved symptoms of reductive stress.
• Cellular respiration was monitored by real-time measurements of CO_2_ and O_2_ fluxes in the gas phase.

## Graphical abstract

VHL-deficiency in renal cells leads to reductive stress characterazied by increased ratio of NADH/NAD^+^. Excess of reducing equivalents (reductive stress) alters ETC and CAC activities. A glucose depletion and supplementation with pyruvate or resazurin, a redox-reactive agent can alleviate reductive stress.

**Figure:**
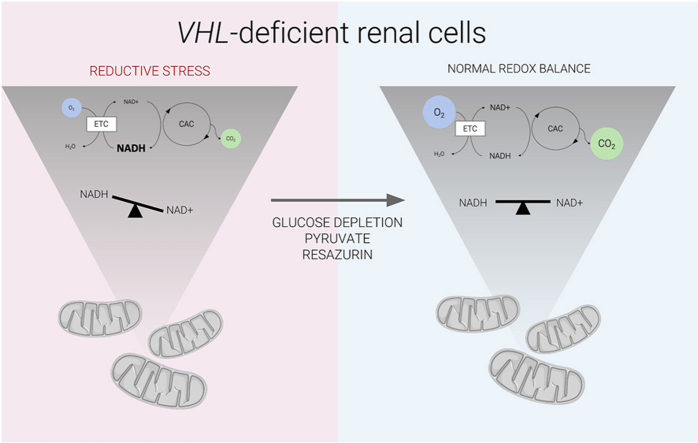

## 1 Introduction

The cellular biology underlying the development of clear-cell renal cell carcinoma (ccRCC) usually involves the inactivation of VHL gene, which is required for normal cellular hypoxia response. VHL disease is a rare hereditary cancer syndrome with a prevalence of up to one in 36,000. The course of VHL syndrome is associated with the development of multiple vascular tumours throughout life, most frequently during young adulthood [[1], [2], [3]]. RCC is the major cause of death among patients with VHL disease, occurring in up to 70% of patients with VHL disease [4]. A series of elegant and groundbreaking studies have shown that VHL tumour suppressor is inactivated in the majority of ccRCC [[5], [6], [7], [8], [9], [10], [11]].

Abnormal or absent VHL protein results in a high level of constitutive hypoxia inducible factor (HIF) expression leading to increased production of VEGF, PDGF, and TGF-α [12,13]. Due in large part to the association of VHL with HIF protein stabilisation, most targeted therapies are directed to inhibit HIF or components of the VEGF pathway [4]. However, given the many essential roles of HIF, shutting down HIF activity is likely to induce intolerable side effects. Since RCC is the major cause of death among patients with VHL disease, which cannot be explained by the proximal effects of HIF signalling alone, resistance to conventional therapies necessitates the need for deeper understanding of underlying mechanisms.

In the present study we used human-derived renal proximal tubular cells (HKC-8 cell line) as a model of normal renal cells and their VHL-deficient counterparts as a model for ccRCC. We show that VHL deficiency leads to reductive stress in the RCC model. Specifically, increased reductive stress in VHL-deficient cells was inferred based on increased NADH/NAD^+^ ratio, decreased respiration and increased accumulation of lipid droplets. Furthermore, VHL-deficient cells appear to be more sensitive to agents that promote aerobic respiration by reducing cellular NADH/NAD^+^ ratio. Together, these results suggest that respiratory activity is suppressed in VHL-deficient cells at least partially due to glucose-associated reductive stress.

Reductive stress is defined as a condition characterized by an excess of reducing equivalents [14]. It will arise in response to conditions that shift the redox balance of important biological redox couples, such as the NAD^+^/NADH, NADP^+^/NADPH, to a more reduced state [14]. Hypoxia-induced reductive stress is known to lead to the accumulation of succinate [15] and l-2-hydroxyglutarate (L2HG), a reduced metabolite of α-KG [14]. Presently, it is still unclear whether and how NADH accumulation affects the metabolic fates of glucose, lipids, and glutamine [14]. To our knowledge, reductive stress in VHL-deficient renal cells has not been described before.

## 2 Results

### 2.1 Aberrant hypoxia response in VHL-deficient renal cells

**Fig. 1:**
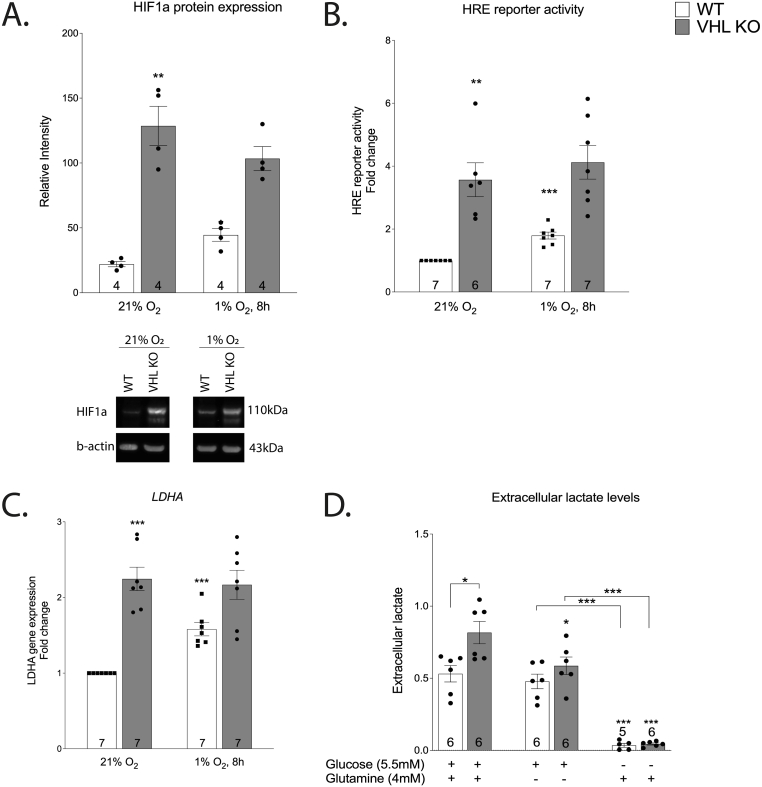
Effect of hypoxia (8 h) on HIF1a protein expression (A), HRE associated transcriptional activity (B) and LDHA gene expression (C) in VHL-deficient cells. The asterisks refer to a statistically significant difference with respect to WT normoxia group unless indicated otherwise. Effect of VHL-deficiency and carbon source availability (4 h) on extracellular lactate levels (D). Asterisks refer to a statistically significant difference with respect to the same genotype on complete medium unless indicated otherwise. Number on the bar indicates sample size. Values are expressed as mean ± SEM. Statistical analysis was performed with unpaired *t*-test with Welch’s correction. *P < 0.05, **P < 0.01, ***P < 0.001.

Human-derived renal proximal tubular cells (HKC-8 cell line) with VHL KO genotype were phenotyped by evaluating the expression and activity of HIF1 and the expression of lactate dehydrogenase A (LDHA) in normoxia and hypoxia. As expected, VHL KO cells exhibited constitutively high HIF1a expression and activity as determined by Western-blot and HRE (hypoxia response element) luciferase reporter assays (Fig. 1A–B). It was parallelled by high LDHA gene expression and high extracellular lactate level in normoxia, suggesting constitutive upregulation of glycolytic activity in VHL KO (Fig. 1C–D). WT cells responded to hypoxia by upregulating HIF1a and LDHA, as expected (Fig. 1A–C). In line with previous studies, these results demonstrate the upregulation of aerobic glycolysis due to the overexpression of HIF1a in VHL-deficient cells and constitutive HIF1a activation in VHL-deficient cells [16,17].

### 2.2 Lower ATP/ADP ratio in VHL-deficient renal cells

**Fig. 2:**
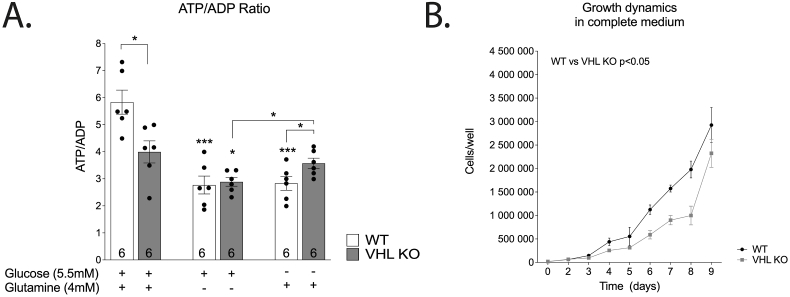
(A) Effect of VHL-deficiency and carbon source availability on ATP/ADP ratio. (B) Effect of VHL-deficiency on culture growth dynamics in the complete medium. Proliferation in glucose- or glutamine-limited medium was severely stunted (limited to 10% confluence). The asterisks refer to a statistically significant difference with respect to the same genotype on complete medium unless indicated otherwise. Number on the bar indicates sample size. Values are expressed as mean ± SEM. Statistical analysis was performed with unpaired *t*-test with Welch’s correction (A) or paired *t*-test (C). *P < 0.05, ***P < 0.001.

In complete medium, ATP/ADP ratio was significantly lower in VHL KO cells when compared to WT (Fig. 2 A). It also coincided with low cellular proliferation rate of VHL KO in complete medium when compared to WT (Fig. 2 B). Removal of either glutamine or glucose from the complete growth medium lowered ATP/ADP ratio. Cell proliferation practically ceased when glucose or glutamine were depleted (Supplementary Fig. S1) indicating that both glutamine and glucose are necessary for normal proliferation of HKC-8 cells. Together, these results suggest impaired ATP production and impaired proliferative capacity in VHL-KO.

### 2.3 ETC and CAC inhibition in VHL-deficient renal cells

**Fig. 3:**
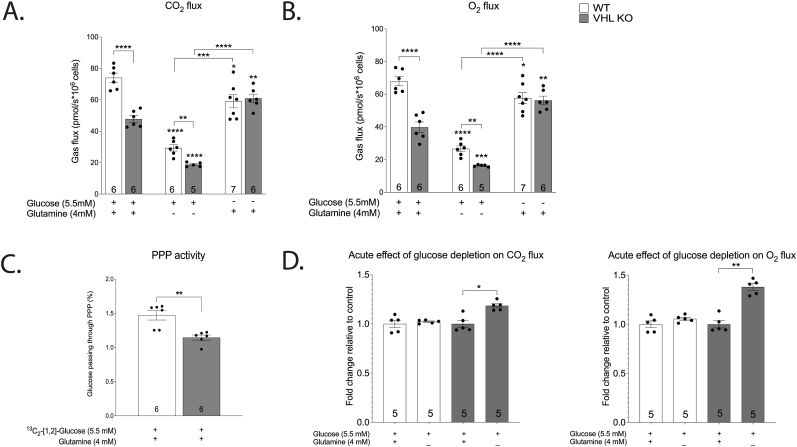
Effect of VHL-deficiency and carbon source availability on respiratory activity based on CO_2_ (A) and O_2_ (B) measurement in gas phase. Results are normalised to cell number based on cyt-c absorbance. Fraction of glucose passing through the pentose phosphate pathway (PPP) based on the ratio of lactate m+1/m+2 isotopes (C). Acute effect of glucose-depletion on the respiration of WT and VHL KO cells (D). To estimate the acute effect of glucose depletion on respiration the mean values of gas flux between complete (before) and glucose-depleted medium (after) were compared using paired *t*-test at 4 time points (25–40 min) after changing the medium. The asterisks refer to a statistically significant difference with respect to the same genotype on complete medium unless indicated otherwise. Number on the bar indicates sample size. Values are expressed as mean ± SEM. Statistical analysis was performed with unpaired *t*-test with Welch’s correction (A–C). *P < 0.05, **P < 0.01, ***P < 0.001, ****P < 0.0001.

Cellular respiration in adherent cells was characterised in terms of O_2_ and CO_2_ fluxes using a cellular gas flux measurement device (please see Methods section for details). Simultaneous monitoring of O_2_ and CO_2_ fluxes offers an overview of the production and aerobic utilization of reducing power in real time, since CO_2_ flux corresponds predominantly to CAC (citric acid cycle) activity and O_2_ flux to ETC (electron transport chain) activity. Another major source of CO_2_ is the oxidative arm of the pentose phosphate pathway (oxPPP). We calculated the fraction of glucose passing through oxPPP based on the ratio of lactate m+1 and m+2 isotopes according to Lee et al. [18]. Isotopic labelling with ^13^C_2_- [1,2]-glucose in normoxic cells indicated modest contribution of PPP to glucose metabolism: 1.47% in WT and 1.15% in VHL KO (Fig. 3 C). Accordingly, in the context of the current study, it was assumed that the CAC is responsible for the overwhelming majority of CO_2_ flux in HKC-8 cells.

Constitutive HIF overexpression led to decreased respiratory activity in the complete medium as evidenced by lower CO_2_ and O_2_ fluxes in VHL KO cells when compared to WT (Fig. 3A–B). In line with previous studies, these results suggest the suppression of CAC and ETC activity in VHL-deficient cells [17].

### 2.4 Respiratory quotient and lipid content

**Fig. 4:**
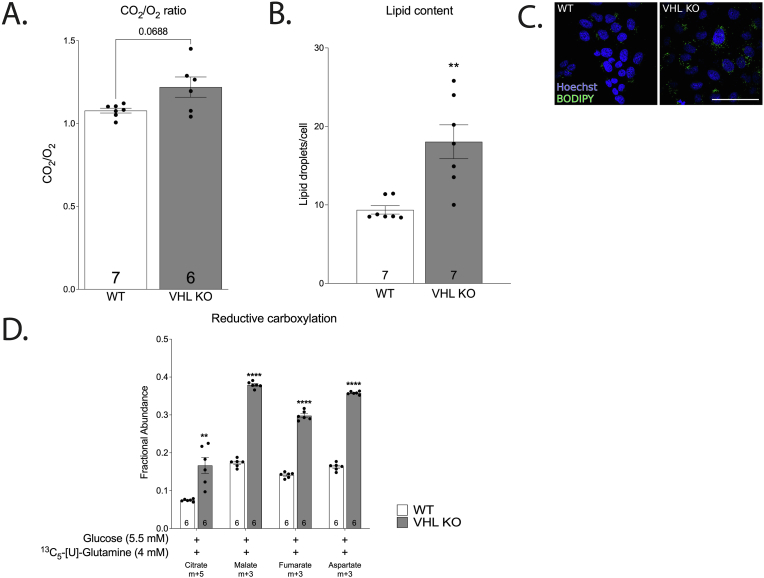
Effect of VHL-deficiency on respiratory quotient (A), lipid content (B) and representative images (C), and reductive carboxylation (D) in complete medium. Representative images of HKC-8 cells stained with Bodipy (stain for neutral lipids) and Hoechst 33258 (stain for nucleic acid) (C). Scale bar equals 100 um. The asterisks refer to a statistically significant difference with respect to WT group unless indicated otherwise. Number on the bar indicates sample size. Values are expressed as mean ± SEM. Statistical analysis was performed with unpaired *t*-test with Welch’s correction. **P < 0.01, ***P < 0.001, ****P < 0.0001.

**Fig. 5:**
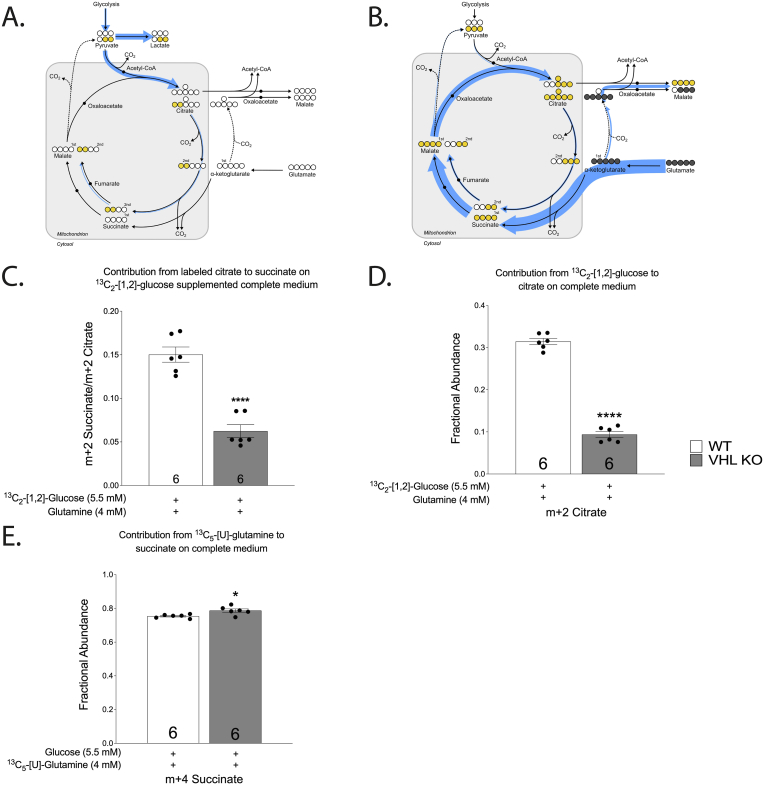
Contribution of ^13^C_2_- [1,2]-glucose (A) or ^13^C_5_-[U]-glutamine (B) to labelled metabolites in complete medium. Thickness of line relates to a percentage of each labelled metabolite. Contribution of labelled citrate to m+2 succinate in ^13^C_2_- [1,2]-glucose (C) supplemented complete medium. Contribution from ^13^C_2_- [1,2]-glucose to citrate (indicated by m+2 isotopomer) on complete medium (D). Contribution from ^13^C_5_-[U]-glutamine to succinate (indicated by m+4 isotopomer) in complete medium (E). The asterisks refer to a statistically significant difference with respect to WT group unless indicated otherwise. Number on the bar indicates sample size. Values are expressed as mean ± SEM. Statistical analysis was performed with an unpaired *t*-test with Welch’s correction. *P < 0.05, ****P < 0.0001.

Respiratory quotient (RQ = CO_2_/O_2_) was above unity in both genotypes with the highest value (RQ ≈ 1.2) in VHL KO cells (Fig. 4 A). RQ > 1 indicates an excess of reductive potential that is not consumed by ETC and suggests that other pathways are actively consuming reducing equivalents in HKC-8 cells. One way to maintain RQ above unity is to channel excess reductive power into fatty acid synthesis [19]. In agreement with this hypothesis, there was a significant increase of lipid droplet accumulation in VHL KO (Fig. 4B–C). There was also a high correlation (Pearson r = 0.89) between RQ and lipid content across genotypes. Stable isotope-mediated nutrient tracing with ^13^C_5_-[U]-glutamine supported upregulation of reductive carboxylation in VHL KO cells based on increased ratio of m+5 citrate and m+3 malate, aspartate and fumarate when compared to WT cells (Fig. 4 D). Furthermore, substantial export of CAC-derived citrate from the mitochondria was supported by metabolomic tracing as evidenced by a modest contribution of labelled citrate to succinate both on labelled glucose and glutamine in both genotypes (Fig. 5A–C). Taken together, these results suggest a more reductive metabolic environment in VHL-deficient renal proximal tubular cells, which is sustained by channelling the excess reductive power into fatty-acid synthesis [20].

### 2.5 Glucose interferes with glutamine-associated energy metabolism in VHL-deficient renal cells

Removal of glutamine reduced respiration in both genotypes by more than 50% compared to the complete medium (Fig. 3A–B). On the other hand, glucose starvation augmented respiration in VHL KO and reduced it in WT resulting overall in similar respiration in both genotypes (Fig. 3A–B). When medium was changed during respiration measurements, the gas flux in VHL KO cells gradually ramped up during a period of approximately 20 min following the transfer from full medium to glucose free medium (Fig. 3 D) and decreased back to the baseline in the same timeframe when transferred back to complete medium (data not shown). In contrast, WT cells did not exhibit significant increase in respiration when transferred from full medium to glucose-free medium (Fig. 3 D). It suggests that VHL-deficiency affects primarily glucose metabolism and to a lesser extent that of glutamine. The finding that glutamine supports higher respiration in HKC-8 cells was supported by metabolomic tracing. Steady state labelling with ^13^C_5_-[U]-glutamine resulted in 75% labelling of the succinate pool in both genotypes (Fig. 5 E) while only ∼15% of succinate originated from m+2 citrate in WT cells on ^13^C_2_- [1,2]-glucose-supplemented complete medium (Fig. 5C)*.* These results suggest that glutamine is the preferred carbon source for cultured HKC-8 cells. Higher respiration in VHL-deficient cells after glucose depletion suggest that increased glycolytic activity limits the contribution from glutamine resulting in reduced CAC and ETC activities in VHL-deficient cells.

**Fig. 6:**
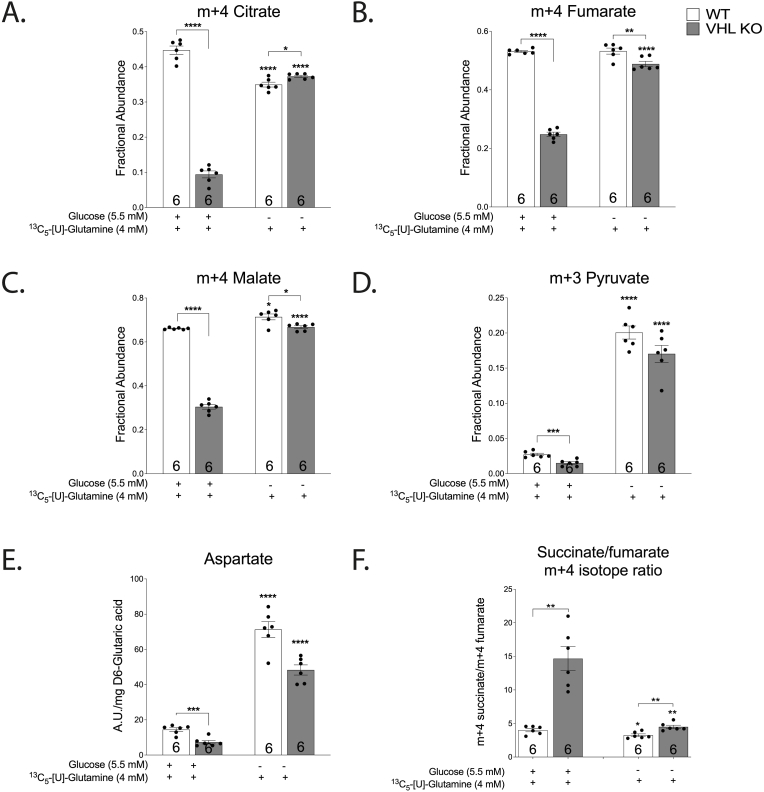
Effect of VHL-deficiency and carbon source availability on m+4 labelled citrate (A), fumarate (B), malate (C), m+3 labelled pyruvate (D), aspartate concentration (E), succinate/fumarate m+4 isotope ratio (F). The asterisks refer to a statistically significant difference with respect to the same genotype on complete medium unless indicated otherwise. Number on the bar indicates sample size. Values are expressed as mean ± SEM. Statistical analysis was performed with unpaired *t*-test with Welch’s correction. *P < 0.05, **P < 0.01, ***P < 0.001, ****P < 0.0001.

Metabolomic tracing with ^13^C_2_- [1,2]-glucose and ^13^C_5_-[U]-glutamine was used to provide insights into the glucose-associated inhibition of respiration in VHL KO. As expected, inhibition of pyruvate decarboxylation was evident in VHL KO cells by the substantial reduction of the citrate pool size (5-fold decrease, Supplementary Fig. S2 C) and in citrate m+2 isotopomer fraction (Fig. 5 D) [16]. Metabolic tracing with glutamine revealed a reduction in the m+4 isotopomer fractional abundance (Fig. 6A–C) and pool sizes of fumarate and malate in VHL KO (Supplementary Figure S2 E-F) when compared to WT in complete medium whereas glutamine contribution to succinate was not reduced (Fig. 5 E). This resulted in a significantly higher ratio of glutamine-derived succinate/fumarate (m+4 isotopomer) in VHL KO on full medium (Fig. 6 F). The high succinate/fumarate ratio in VHL KO was markedly reduced by glucose-starvation (Fig. 6 F), elevating both fumarate and malate closer to WT levels (m+4 isotopomer; Fig. 6B–C). Simultaneously, glucose depletion resulted in higher CO_2_ flux in VHL KO cells when compared to complete medium (Fig. 3 A).

Although in glucose-depleted medium the citrate pool was lower in VHL KO cells (Supplementary Fig. S2 C), the contribution of glutamine to the citrate pool (m+4 isotopomer) was similar to WT in glucose depleted medium (Fig. 6 A). When glutamine was the sole carbon source, most pool sizes of oxidative CAC intermediates were smaller in HKC-8 renal cells (Supplementary Figure S2 C-F) except for the aspartate pool size which was remarkably larger than in complete medium (Fig. 6 E). This suggests that, on glutamine, the oxidative pathway predominantly terminates at the oxaloacetate to form aspartate instead of operating as a cycle to yield citrate. Glucose-depletion also activates glutamine contribution to pyruvate, presumably through the malic enzyme, as seen from the nearly tenfold increase of m+3 pyruvate fraction in both genotypes (Fig. 6 D). Together, these findings suggest a shift towards a more oxidative metabolism in response to glucose depletion in VHL-deficient cells.

### 2.6 VHL-deficient cells exhibit glucose-associated reductive stress

We hypothesised that the observed suppression of CAC activity might be at least partly due to a higher level of reductive stress experienced by VHL KO cells and, hence, could be ameliorated by reducing the availability of cellular reducing equivalents. For example, cells can reduce cytosolic NADH/NAD^+^ ratio by reducing exogenous pyruvate to lactate and then expelling lactate from the cell. In general, a high NADH/NAD^+^ ratio is expected to slow down the operation of CAC due to product inhibition. Therefore, suitable electron acceptors might be able to lower reducing potential in cells as long as they accept electrons from common reducing equivalents such as NADH and CAC organic acid intermediates.

In order to test the hypothesis that the suppression of CAC activity in VHL KO is at least partially due to elevated cellular reductive potential, we applied three agents with different modes of action, but all potentially capable of reducing cellular NADH/NAD^+^ ratio. These agents were the uncoupler dinitrophenol (DNP), pyruvate and resazurin. DNP promotes the leakage of protons from mitochondrial intermembrane space to the lumen, leading to increased consumption of mitochondrial NADH in order to maintain the mitochondrial membrane potential. Pyruvate can lead to the consumption of cytosolic NADH by the LDH reaction whereby pyruvate is reduced to lactate. Resazurin is a cell-permeable redox-sensitive dye that can be irreversibly reduced to pink resorufin while resorufin can itself undergo further reversible reduction to translucent dihydroresorufin [21].

**Fig. 7:**
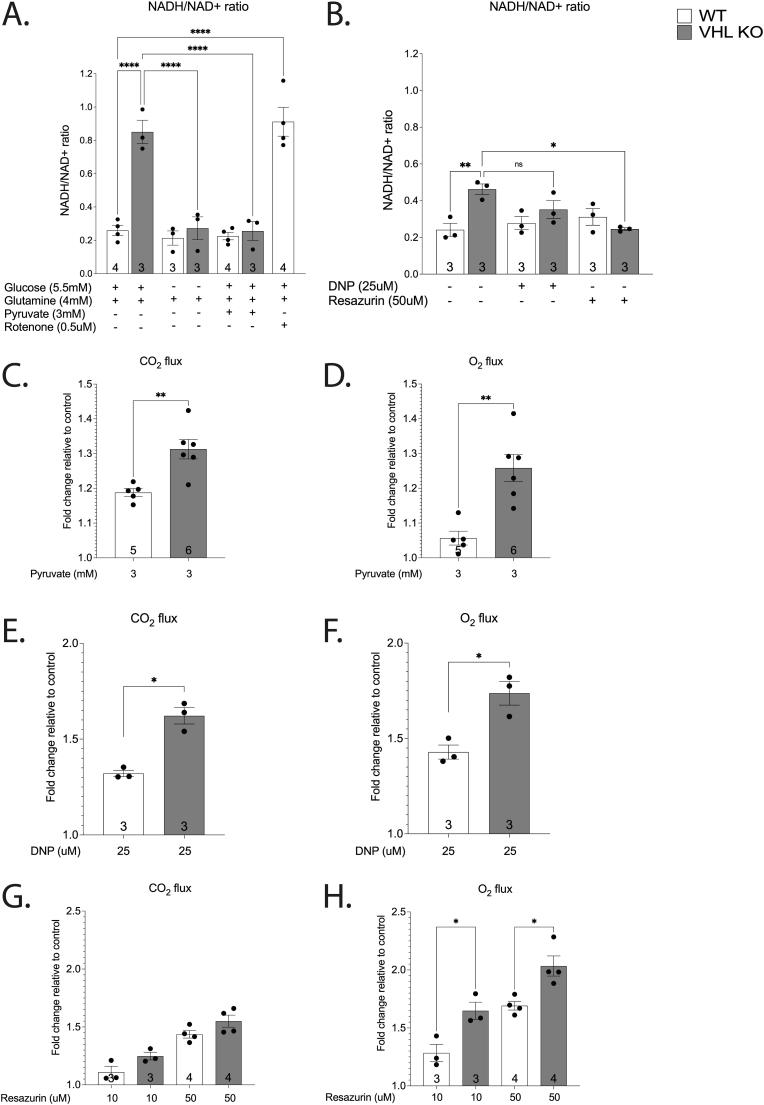
NADH/NAD^+^ ratio (A–B) in response to glucose depletion (4 h), pyruvate (3 mM, 4 h), resazurin (50 uM, 1h), or DNP (25 uM, 1h) in WT and VHL KO HKC-8 cells. O_2_ and CO_2_ fluxes in response to pyruvate (3 mM) (C–D), uncoupler dinitrophenol (DNP) (25 uM) (E–F) or resazurin (50 uM) (G–H) in WT and VHL KO HKC-8 cells. Results are normalised relative to the control group (C–H). Number on the bar indicates sample size. Values are expressed as mean ± SEM. Statistical analysis was performed with One-way ANOVA with post-hoc Tukey HSD Test (A, B, G, H) or unpaired *t*-test with Welch’s correction (C–F). *P < 0.05, **P < 0.01, ****P < 0.0001.

**Fig. 8:**
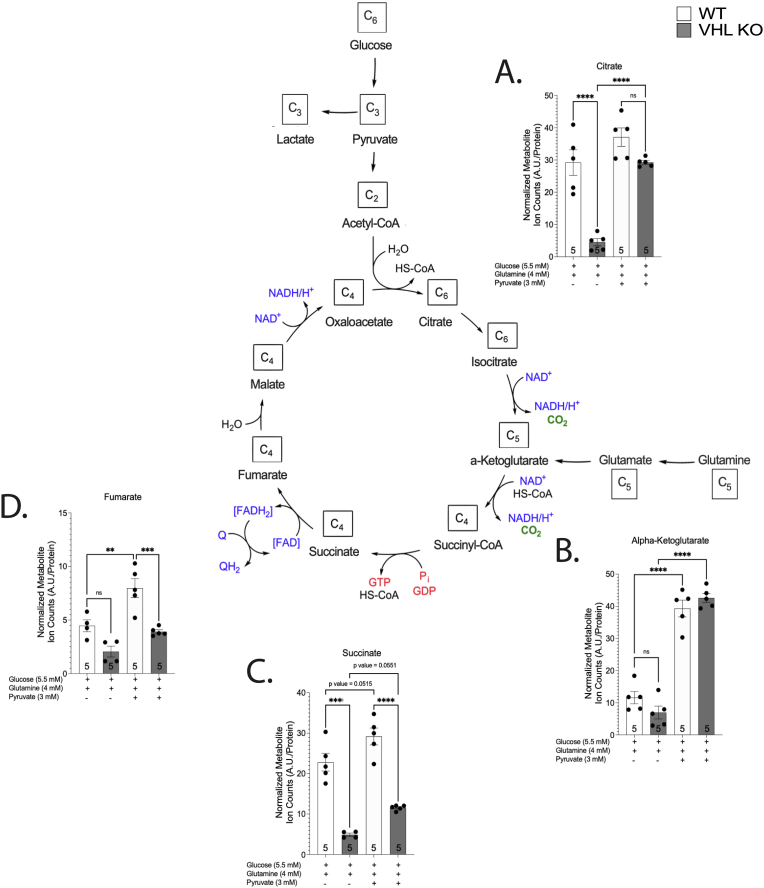
Effect of VHL-deficiency and pyruvate supplementation on metabolite pool sizes. Number on the bar indicates sample size. Values are expressed as mean ± SEM of normalised metabolite ion counts. Statistical analysis was performed with One-way ANOVA with post-hoc Tukey HSD Test. **P < 0.01, ***P < 0.001, ****P < 0.0001.

VHL KO cells exhibited reductive stress as evidenced by a two-to four-fold increase in the NADH/NAD^+^ ratio (Fig. 7A–B) when compared to WT cells. This effect was glucose dependent, as the depletion of glucose normalised NADH/NAD^+^ ratio in VHL KO cells (Fig. 7 A). Reductive stress was also relieved by pyruvate and resazurin treatments, but not rotenone (negative control) (Fig. 7A–B). The effect of DNP on NADH/NAD^+^ ratio was not significant (Fig. 7 B). In line with our hypothesis, VHL KO cells were more susceptible to the stimulation of respiration by pyruvate, DNP and resazurin as evidenced by a significantly higher induction of respiration when compared to WT cells (Fig. 7C–H). This effect was especially pronounced on O_2_ flux after treatment with 3 mM pyruvate (Figs. 7 D) and 10 μM resazurin (Fig. 7H). Metabolomic analysis confirmed the pyruvate-induced activation of CAC in VHL KO as evidenced by the markedly increased pool size of citrate in pyruvate-treated VHL KO cells (Fig. 8A). Together, these results suggest that respiratory activity is suppressed in VHL KO cells at least partially due to glucose-associated reductive stress.

## 3 Discussion

It is well established that VHL loss-driven HIF stabilisation in ccRCC leads to the impairment of mitochondrial respiration, a switch to aerobic glycolysis [17] and lipid deposition in ccRCC [20]. Highly upregulated HIF activates the transcription of PDK1 gene (pyruvate dehydrogenase kinase), which inactivates PDH (pyruvate dehydrogenase), the mitochondrial enzyme that converts glucose derived pyruvate to acetyl CoA for entry into the tricarboxylic acid [22] thus inhibiting normal oxidative phosphorylation. Further, upregulation of HIF results in increased reductive carboxylation of glutamine due to the inhibition of alpha-ketoglutarate dehydrogenase by the E3 ubiquitin ligase SIAH2 [23].

In accordance with previous reports from VHL-deficient tumours [[24], [25], [26]], we show that VHL-deficient renal cells undergo metabolic shift to aerobic glycolysis, characterised by impaired respiration and increased levels of glycolysis, as measured by decreased oxygen consumption, decreased CO_2_ production and increased extracellular lactate levels. As a novel finding, we provide evidence that VHL-deficiency is associated with glucose-induced reductive stress that is accompanied by impaired cellular respiration, impaired CAC activity, upregulation of reductive carboxylation of glutamine and accumulation of lipid droplets in VHL-deficient cells.

Reductive stress in VHL-deficient cells was established based on increased NADH/NAD^+^ ratio that is associated with reduced normoxic respiration when compared to WT cells. Reduced respiration in the context of increased cellular NADH/NAD^+^ ratio suggests an impaired capacity of ETC to accept electrons since most of the cellular NAD(H) pool resides in the mitochondrion [27].

By removing glucose, reductive stress was mitigated in VHL KO cells as evidenced by a comparable NADH/NAD^+^ ratio with WT and increased cellular respiration in glucose-free medium. We hypothesised that glucose was inhibiting respiration by contributing to the accumulation of reducing equivalents. In support of this hypothesis, pyruvate and resazurin were found to relieve reductive stress in VHL-deficient cells with concomitant activation of CAC activity and cellular respiration. Thus by targeting the NADH pool we were able to normalise NADH/NAD^+^ ratio and increase respiration in VHL KO cells.

The concept of reductive stress, which has languished in scientific research for decades has experienced a surge in popularity recently due to coverage in prestigious journals [15,[28], [29], [30]]. Ghyczy and Boros [28] define reductive stress as “… an abnormally increased electron pressure or reducing power and it can occur either as a result of pathological processes leading to an excess of high-energy reducing electrons (as in NADH), a failure of mechanisms available for dealing with this rise in electron pressure, or, occasionally perhaps, a combination of both.” In a related commentary, Lipinski [29] mentions that many degenerative diseases are associated with a hypoxic state that results in an increased NADH/NAD^+^ ratio, leading to a reductive cytosolic environment [30]. Glycolysis and the CAC produce NADH, which provides electrons for mitochondrial ETC to produce ATP. In certain scenarios, it is possible that there is an over-production of reducing equivalents resulting in reductive stress. During limited food supply or extreme physical activity it is expected that ETC will consume nearly all of the reducing power produced by CAC in order to ensure an adequate supply of cellular ATP. In modern times, however, an opposite scenario is much more likely. Overconsumption of nutrients (especially sugars) powers up the CAC to produce an excess amount of reducing equivalents that are not consumed by the ETC (e.g. due to a lack of physical activity) and must be disposed of in other ways. This necessitates a shift away from cells normal metabolism creating reductive stress. During ischemic stroke, for example, ATP synthesis in inhibited due to the lack of oxygen leading to a buildup of succinate, which upon reoxygenation will initiate reverse electron transfer from complex II to I and trigger a massive production of superoxide resulting in ischemia-reperfusion injury [15]. As such, there have evolved cellular mechanisms to mitigate the dangers posed by reductive equivalents. One of those is the transport of citrate from the CAC to cytoplasm where it is converted to fatty acids and stored as lipid droplets [20], which are chemically more stable depos of reductive power. Another mechanism could be the activation of antioxidant response to counteract oxidative stress downstream of reductive stress [31]. Finally, reductive stress and related coping mechanisms are also relevant for tumour biology, since tumours tend to thrive in hypoxic environments and rely on glycolytic activity to compensate for the lack of CAC and ETC activity.

As the current study identifies aspects of the VHL KO phenotype that relate to reductive stress, further studies in animal models (e.g. VHL KO mice) and patient samples would be needed to assess the relevance of reductive stress in VHL-deficient tissues and its relation to clear cell renal carcinoma. For example, cells from patients with VHL-deficient tumours could be used to assess whether the general findings observed here apply broadly across the syndrome. Furthermore, it would be worthwhile to study reductive stress in vivo, in VHL-deficient mice for example, to understand how well a gene knockout model replicates the findings reported here. We expect that the current study provides several important directions for translational studies of the metabolic phenotype of VHL-deficient renal cells.

## 4 Conclusion

Understanding the mechanisms of RCC tumorigenesis is important to further develop strategies and treatment options. We have demonstrated increased reductive stress in the RCC model that is accompanied by impaired cellular respiration, impaired CAC activity, upregulation of reductive carboxylation of glutamine and accumulation of lipid droplets in VHL-deficient cells. Manipulation of the cytosolic NADH pool resulted in enhanced respiration and normalisation of NADH/NAD^+^ ratio. Future studies are needed to further determine whether and how reductive stress affects the metabolism of glucose, lipids, and glutamine in VHL-deficient tumour tissues.

## 5 Material and methods

### 5.1 Cell lines

Wild-type, CRISPR-generated VHL knock-out human kidney proximal tubular (HKC-8) cells were a gift from Professor Sir Peter J. Ratcliffe. HKC-8 cells were cultured in minimum essential medium (MEM) (Capricorn, MEM-STA) supplemented with 10% fetal bovine serum (FBS) (Gibco™, 10270106) and 100 U/ml penicillin and 100 ug/ml streptomycin (Gibco™, 15140122). Cells were grown in an incubator with 5% CO_2_ at (Gibco™, 15140122)37 °C under atmospheric oxygen concentration (21% O_2_). For hypoxia experiment (1% O_2_, 8 h), a modular incubator chamber (Billups-Rothenberg inc, MIC-101) was used. Cells were placed in the chamber, a flow meter was attached to the unit and the chamber was flush with 20l of 1% O_2_, 5% CO_2_, 94% N_2_ gas mixture. The chamber was sealed and placed in a 37 °C incubator.

### 5.2 Luciferase-reporter assay

HKC-8 cells were plated at a density of 60,000/well in a 12-well plate. Next day, cells were co-transfected with 187.5 ng pGL4.42[luc2P/HRE/Hygro] (Promega, E4001) and 12.5 ng pRL-TK (Promega, E2241) luciferase vectors by Effectene transfection reagent (Qiagen, 301425). 24 h later, the cells were exposed to normoxia (21% O_2_) or hypoxia (1% O_2_) for 8 h. Cells were lysed in 1x passive lysis buffer (Promega, E1941) with gentle shaking at RT for 15 min. Firefly & Renilla Luciferase Single Tube Assay (Biotium, 30081) was performed according to the manufacturer's protocol. Luminescence was measured on a Promega GloMax Multi Plus Plate Reader using a 10-s pre-read delay, followed by a 5-s measurement period.

### 5.3 Western blotting

HKC-8 cells were plated at a density of 80,000/well in a 12-well plate. Next day, cells were exposed to normoxia (21% O_2_) or hypoxia (1% O_2_) for 8 h. Lysates were used for the detection of HIF1a protein. Cells were lysed in 1x passive lysis buffer (Promega, E1941) supplemented with 1x protease inhibitor (ThermoFisher Scientific, 78430) with gentle shaking at 4 °C for 30 min. Protein concentration was determined with the BCA method (Pierce BCA Protein Assay Kit, ThermoFisher Scientific, 23225) according to the manufacturer's protocol. Whole cell lysates were obtained, electrophoresed on gel and transferred as previously described (Eskla et al., 2018). Immunoblots were probed with rabbit anti–HIF–1α (1:2000) (Novus, NB100–479) and mouse anti-β-actin (1:1000) (Santa Cruz Biotechnology, sc-47778). β-actin was used as a loading control. Fluorescent conjugated secondary antibodies goat anti-rabbit antibody (Jackson ImmunoResearch, 35569) and goat anti-mouse antibody (Invitrogen, A21057) were used. Immunoblots were imaged on a LI-COR Odyssey CLx system (LI-COR Biotechnologies). Images were converted to grayscale and band intensities were quantified in Image Studio Lite v 3.1.4 (LI-COR Biotechnologies).

### 5.4 ADP/ATP Ratio Assay Kit

HKC-8 cells were plated at a density of 10,000 cells in a 96-well luminometer-compatible tissue culture plate. Next day, cells were washed with PBS and grown in DMEM (ThermoFisher, A1443001) supplemented with 10% FBS, 1x Penicillin/Streptomycin and various carbon sources for 4 h (1) Complete medium contained 5.5 mM glucose (Gibco™, A2494001) and 4 mM glutamine (Sigma-Aldrich, G7513), (2) glutamine free medium contained only 5.5 mM glucose and (3) glucose free medium contained only 4 mM glutamine. Ratio of ATP to ADP was measured with ADP/ATP Ratio Assay Kit (Sigma-Aldrich, MAK135-1 KT) according to the manufacturer's protocol. Luminescence was measured using a Promega GloMax Multi Plus Plate Reader.

### 5.5 NAD^+^/NADH Ratio Assay Kit

Total cellular NADH and NAD^+^ levels were determined using the EnzyChrom NAD^+^/NADH assay kit (BioAssay Systems, E2ND-100). Briefly, HKC-8 cells were plated at a density of 400,000 cells in a 6-well plate. Next day, cells were washed with PBS and grown in DMEM (ThermoFisher, A1443001) supplemented with 10% FBS, 1x Penicillin/Streptomycin and various carbon sources for 4 h (1) Complete medium contained 5.5 mM glucose (Gibco™, A2494001) and 4 mM glutamine (Sigma-Aldrich, G7513), (2) glutamine free medium contained only 5.5 mM glucose and (3) pyruvate supplemented medium contained, 5.5 mM glucose, 4 mM glutamine and 3 mM sodium pyruvate (Gibco™, 11360070). DNP (25 uM) (Sigma-Aldrich, D198501) and resazurin (10 uM or 50 uM) (Sigma-Aldrich, R7017) treatments were performed for 1 h. Cells were washed with PBS. NAD^+^ and NADH were extracted from the lysate according to the manufacturer’s protocol. The change in absorbance at 565 nm for 15 min at room temperature was measured.

### 5.6 Quantitative real-time reverse transcription PCR

HKC-8 cells were plated at a density of 300,000/well in a 6-well plate. Next day, cells were exposed to normoxia (21% O_2_) or hypoxia (1% O_2_) for 8 h followed by extraction of RNA with TRIzol reagent (MRC, TR 118) according to the manufacturer's protocol. Reverse transcription was performed using one ug of total RNA with random hexamers (LGC Biosearch Technologies) and SuperScript III Reverse Transcriptase (Thermo Fisher Scientific, 18080044). Quantitative real-time RT-PCR gene expression studies were conducted as previously described [32]. Primers were designed for the detection of *LDH-A* and *HPRT* (housekeeper) transcripts (LGC Biosearch Technologies). Real-time qPCR was performed using HOT FIREPol EvaGreen qPCR Supermix (Solis BioDyne, 08-36-00001). qPCR reactions were run on QuantStudio 12 K Flex Software v.1.2.2 Real-Time PCR System equipment (Applied Biosystems, USA) and quantified with the QuantStudio 12 K Flex Software v.1.2.2.

Following primers were used for qPCR analysis:

*LDH-A* Forward Primer (5’3’) GCTGGGAGTTCACCCATTAAGCTG.

*LDH-A* Reverse Primer (5’3’) CAATAGCCAGGATGTGTAGCCTTTGAG.

*HPRT1* Forward Primer (5’3’) GACTTTGCTTTCCTTGGTCAGG.

*HPRT1* Reverse Primer (5’3’) AGTCTGGCTTATATCCAACACTTCG.

### 5.7 Growth curve

HKC-8 cells were plated at a density of 50,000/well in 2 ml culture medium in 6-well plate. Two replicates were counted every 24 h for 7 days by EVE Automated Cell Counter (NanoEnTek).

### 5.8 Lipid content

Cells were plated at a density of 25 000/well onto 8-well culture slides (Falcon®, 354118). Following day, cells were fixed in 4% paraformaldehyde/phosphate buffered saline (PBS) solution at 36 °C over 15 min. After washing with PBS five times over 5 min each cell was stained with BODIPY 493/503 dye (1uM, Invitrogen, D3922) in PBS over 10 min and rinsed thrice with PBS over 5 min. BODIPY 493/503 can be used to label cellular neutral lipid contents. Subsequently, nuclei were stained with 5 ug/ml Bisbenzimide H 33258 (Hoechst 33258, Sigma Aldrich) in PBS for 5 min, washed with ddH_2_O and mounted in Fluoromount (Sigma Aldrich) mounting media.

### 5.9 Microscopy and image analysis

Fluorescent images were obtained with Olympus FV1200MPE (Olympus, Germany) laser scanning confocal microscope (objective lens: Olympus^TM^ 60X Oil objective, PlanApo, 1.42NA/0.15WD). Near Violet Laser Diode (LD405, 50 mW, Olympus); Sepia Laser Diode (LD473, 15 mW, Olympus) lasers were used in combination with U-MNU2 and UMWIBA3 filter cubes (Olympus) for fluorescence detection. Seven independent experiments were conducted. From each replica three non-overlapping fields of view (211.97 x 211.97 um) were acquired. Images were analysed using Fiji software [33]. Lipid droplets were counted using the “Find Maxima” tool (prominence >15). Cell nuclei (cell count) were counted with the “Analyze Particles” tool (images converted to 8 bit format, threshold values set 1–255, analysed particle size: 100-Infinity um^2^).

### 5.10 Lactate levels

HKC-8 cells were plated at a density of 450,000/well in a 6-well plate. Next day, cells were washed with PBS and grown in DMEM (ThermoFisher, A1443001) supplemented with 2% FBS, 1x Penicillin/Streptomycin and various carbon sources for 4 h (1) Complete medium contained 5.5 mM glucose and 4 mM glutamine, (2) glutamine free medium contained only 5.5 mM glucose, (3) glucose free medium contained only 4 mM glutamine. 100 ul of medium was collected and stored at −80 °C. Targeted metabolomics was carried out with selected hydroxyl acids using a previously described method [34]. For analysis of hydroxy acid and hexoses, 50 ul of the sample was mixed with 30 ul (500 uM [2H4]-succinic acid in methanol). The samples were centrifuged for 15 min at 10000×*g* and 20 ul were analysed by liquid chromatography-mass spectrometry QTRAP 4500 (AB Sciex, Canada).

### 5.11 Isotope labelling

For the isotope labelling experiments, HKC-8 cells were plated at a density of 400,000/well in a 6-well plate. Next day, cells were washed with PBS and grown (atmospheric oxygen, CO_2_-free at 37 °C) in DMEM (Sigma-Aldrich, D5030) supplemented with 20 mM HEPES (Sigma-Aldrich, H0877) and various carbon sources for 4 h (1) Complete medium (labelled glutamine) contained 5.5 mM glucose and 4 mM ^13^C_5_-[U]-glutamine (Cambridge Isotope Laboratories Inc., CLM-1822-H). (2) Complete medium (labelled glucose) contained 5.5 mM ^13^C_2_- [1,2]-glucose (Cambridge Isotope Laboratories Inc., CLM-504-1) and 4 mM glutamine. (3) Glucose free medium contained only 4 mM ^13^C_5_-[U]-glutamine. Pyruvate supplemented medium contained 5.5 mM glucose, 4 mM glutamine and 3 mM ^13^C_3_-[U]-sodium pyruvate (Cambridge Isotope Laboratories Inc., CLM-2440). After incubating 4 h, cells were placed on ice and 800 ul of medium was collected and stored at −80 °C. Cells were then washed with ice-cold saline. Next, cells were covered with 500 ul of ice-cold methanol (Sigma-Aldrich). 200ul of milliQ supplemented with 1 ug of D6-glutaric acid was added and set on ice for approximately 1 min. Cells were scraped and collected in a precooled 1.5 ml tube. 500 ul of chloroform was added to each sample. Tubes were vortexed and left on a shaker for 15 min at 4°. Next, samples were centrifuged at 13 000 rpm for 5 min forming two layers from which the upper, methanol layer with polar metabolite was collected and stored at −80 °C until GC-MS. Before GC-MS samples were dried with aeration (37 °C, N_2_ wind).

GC-MS analysis was undertaken using an Agilent 7890B GC and 5977A MSD. 1 ul of sample was injected in splitless mode with helium carrier gas at a rate of 1.0 ml min^−1^. Initial oven temperature was held at 100 °C for 1 min before ramping to 170 °C at a rate of 10 °C min^−1^, followed by a ramp to 220 °C at a rate of 3 °C min^−1^ and a final ramp to 300 °C at a rate of 10 °C min^−1^ with a 9 min hold. Compound detection was carried out in single ion monitoring (SIM) mode. Total ion counts of each metabolite were normalised to the internal standard D6-glutaric acid (Qmx Laboratories Ltd, D-5227).

### 5.12 Cells respiration experiments

For the cell respiration measurements, HKC-8 cells were seeded at a density 8x10^6^ on a gelatin-coated sterile glass disc (Pilkington Microwhite) placed in the bottom of a 100 mm dish. Next day, cells were washed with PBS and grown (atmospheric oxygen, CO_2_-free at 37 °C) in bicarbonate-free DMEM (Sigma-Aldrich, D5030) buffered with 20 mM HEPES and various carbon sources for 1 h. Complete medium contained 5.5 mM glucose and 4 mM glutamine, (2) glutamine free medium contained only 5.5 mM glucose, and (3) glucose free medium contained only 4 mM glutamine. To estimate acute effect of glucose depletion, DNP, pyruvate or resazurin, cells were washed with PBS and placed in bicarbonate-free complete medium buffered with 20 mM HEPES for 1 h.

Before the respiration experiment, 25 ug/ml carbonic anhydrase (Sigma-Aldrich, C2624) was added to the medium. Cellular respiration was recorded by a novel cellular gas flux measurement device developed in our lab [35]. The system allows to determine O_2_ and CO_2_ fluxes in adherent cell cultures in real time and measure the production (by CAC) and utilization (by ETC) of reducing power. Optical absorption measurements were performed before and after the respiration experiment (except for Resazurin). Respiration was normalised to cell number based on cyt-c absorbance.

Next, the medium was poured off and the disc was placed in the respiration chamber. To prevent the cells from drying out, 0.5 ml medium was pipetted on the disc. Chamber was closed and the system was switched to high throughput mode for 15 s to equilibrate the gases in the system. To estimate acute effect of glucose depletion, DNP, pyruvate or resazurin, the glass disc was removed from the respiration chamber, medium was allowed to drain off, and the disc was placed into complete or glucose free medium either supplemented with DNP (25 or 100 uM) (Sigma-Aldrich, D198501), sodium pyruvate (3 mM) (Gibco™, 11360070), resazurin (10 or 50 uM) (Sigma-Aldrich, R7017) or left untreated. Glass disc was shaken gently to allow the new medium to diffuse. Next, the disc was placed back in the respiration chamber.

## Author contribution

HV, KLE and HL conceived the study and wrote the manuscript with assistance from DAT, VV and EV. HV, KLE and AH conducted cell culture experiments. HV, AH, and HE performed cellular gas flux measurements. KLE and AH performed molecular biology studies. RP conducted lactate measurement. AL and VO designed the cellular gas flux measurement system. HR built the cellular gas flux measurement system. TJ performed lipid staining. DAT, AT and JR performed GC-MS experiments.

## Declaration of competing interest

The authors declare that they have no known competing financial interests or personal relationships that could have appeared to influence the work reported in this paper.
