# Supplementary material for: VHL-deficiency leads to reductive stress in renal cells
Source: Free Radic Biol Med. 2023 Nov 1;208:1–12. doi: 10.1016/j.freeradbiomed.2023.07.029 (PMC10602395; doi:10.1016/j.freeradbiomed.2023.07.029)

Supplementary Figure S1.

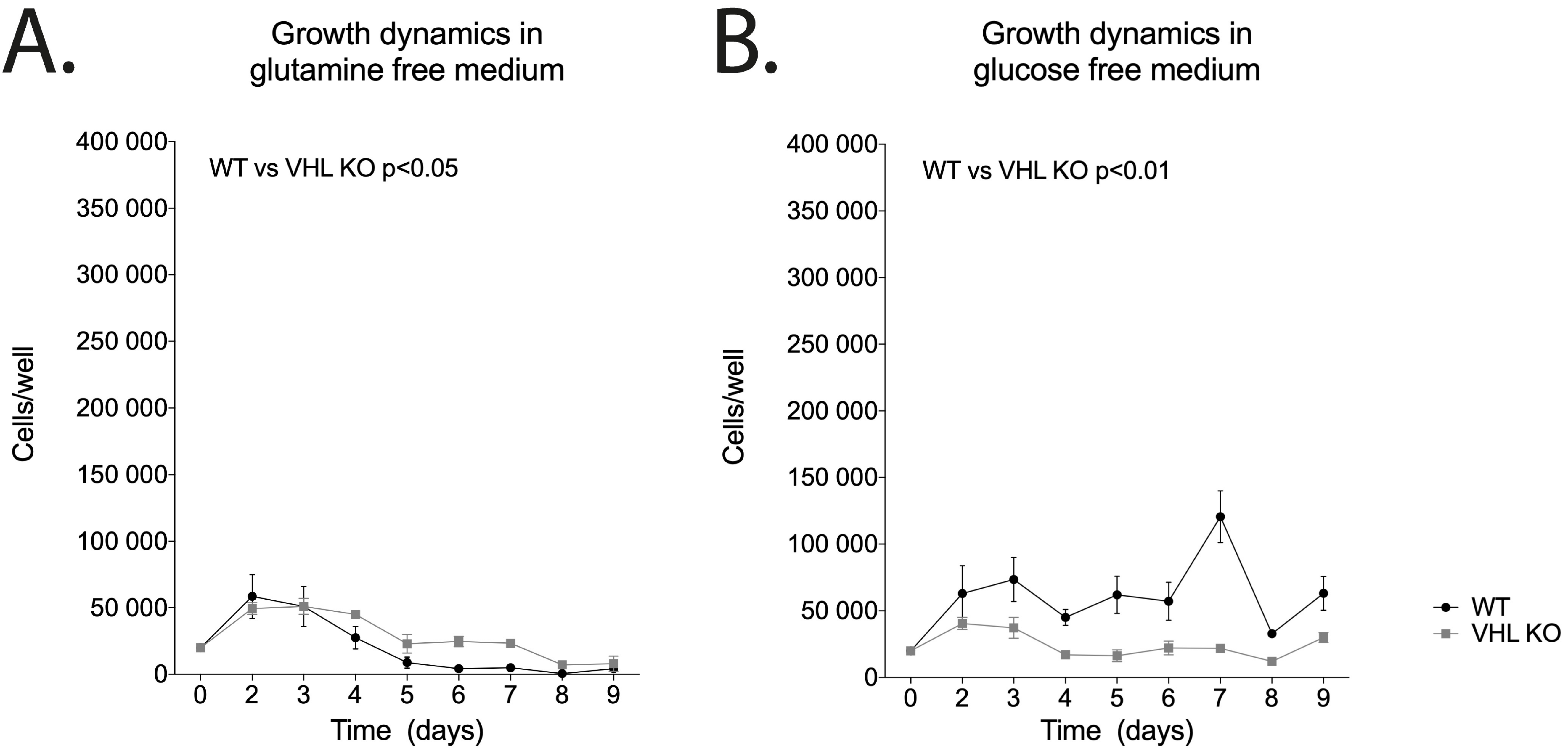

Supplementary Figure S2.

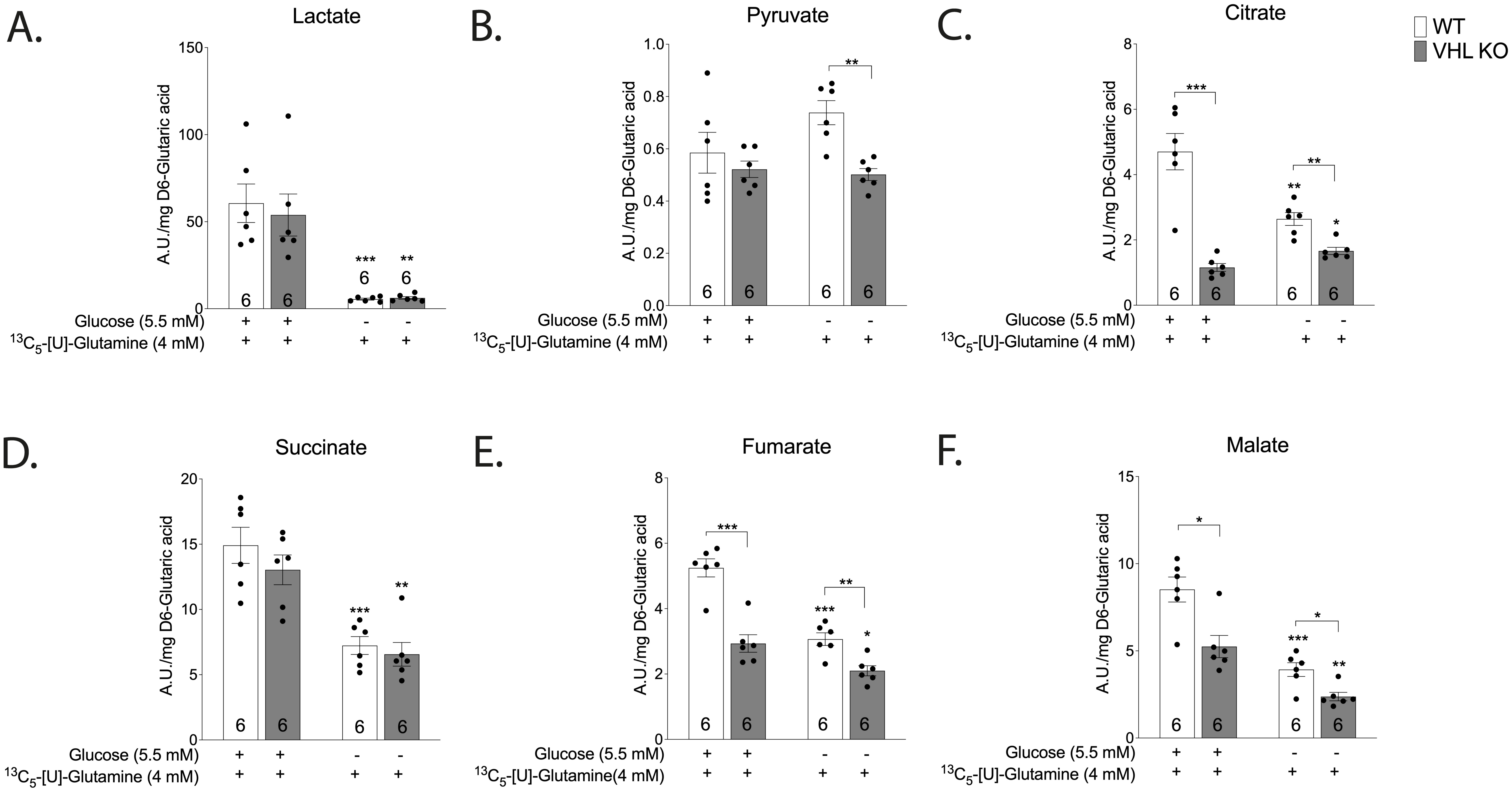

Supplement: Multimedia component 1 [file mmc1.pdf]
